# Hormetic effect of 17α-ethynylestradiol on activated sludge microbial community response

**DOI:** 10.3389/fmicb.2022.961736

**Published:** 2022-08-18

**Authors:** Phumudzo Budeli, Mutshiene Deogratias Ekwanzala, Maggy Ndombo Benteke Momba

**Affiliations:** ^1^Department of Environmental, Water and Earth Sciences, Tshwane University of Technology, Pretoria, South Africa; ^2^Department of Infectious Diseases, Institute of Biomedicine, University of Gothenburg, Gothenburg, Sweden

**Keywords:** Hormesis, EE2 (17α-ethynylestradiol), activated sludge microbiome, metagenomics

## Abstract

Synthetic estrogen analogues are among the most potent estrogenic contaminants in effluents from wastewater treatment plants. Although its effects have been well elucidated in the feminization of male fish and interference with the endocrine systems in humans, it has not been fully explored in the activated sludge (AS) microbiome, particularly EE2 (17α-ethynylestradiol). Therefore, in this study, the bacterial community shift in a 6-day laboratory-scale reactor in environmental (0, 5, 10, and 100 ng/L) and predictive elevated concentrations (5, 10, and 100 mg/L) of EE2 was investigated using culture-based and metagenomics approaches. Results showed significant changes (*t*-test, all *p* < 0.05) between initial and final physicochemical parameters (pH, DO, and EC). Although environmental concentrations showed a slight decrease in microbial counts (5.6 × 10^6^ to 4.6 × 10^6^ CFU/ml) after a 24-h incubation for the culturable approach, the predictive elevated concentrations (5 to 100 mg/L) revealed a drastic microbial counts reduction (5.6 × 10^6^ to 8 × 10^2^ CFU/ml). The metagenomic data analysis uncovered that bacterial communities in the control sample were dominated by *Proteobacteria*, followed by *Bacteroidetes* and *Firmicutes*. The taxonomic classification after exposure of microbial communities in various concentrations revealed significant differences in community composition between environmental concentration (Shannon indices between 2.58 to 3.68) and predictive elevated concentrations (Shannon indices between 2.24 and 2.84; *t*-test, all *p* < 0.05). The EE2 enriched seven OTUs were *Novosphingobium, Cloacibacterium, Stenotrophomonas, Enterobacteriaceae*_unclassified, *Stenotrophomonas, Enterobacteriaceae*_unclassified and *Rhodobacteraceae*_unclassified. These results were supported by a dehydrogenase activity (DHA) test, which demonstrated less (about 40%) DHA in predictive elevated concentrations than in environmental concentrations. Notwithstanding, these findings suggest that EE2 may possess potent hormetic effect as evidenced by promotion of microbiome richness and dehydrogenase activity of AS in lower EE2 doses.

## Introduction

Endocrine-disrupting compounds (EDCs) are a class of micropollutants that have been detected in the environment since the 1930s (Rahman et al., 2009; Cook et al., 2016). These compounds have recently attracted much attention due to their detrimental effects on the endocrine system in humans and the environment. Previous investigators have pointed out that natural estrogens such as estrone (E1), 17β-estradiol (E2), and estriol (E3) and synthetic estrogens such as 17α-ethinylestradiol (EE2) have higher potential for endocrine-disrupting effects (Adeel et al., 2017; Amin et al., 2018; Beck et al., 2018). Other studies have shown that EE2 is the most persistent in the environment; among estrogen classes, some natural estrogens can degrade spontaneously (Lai et al., 2002; Zhang et al., 2016). According to Ting and Praveena (2017), a woman’s body only utilizes 20% of the birth oral contraceptive pills. The main ingredient is EE2; the remaining 80% is passed down to the toilets through excretion as non-metabolized conjugates (Yang et al., 2011).

Previous studies have identified wastewater treatment plants (WWTPs) as the main sinks of synthetic estrogenic compounds (Zhang et al., 2016; Kibambe et al., 2020). However, these compounds reach the WWTPs through waste discharge from domestic toilets, pharmaceutical industries and hospitals (Li et al., 2011; Silva et al., 2012; Ting and Praveena, 2017). Pharmaceutical industries are the principal sources of complex non-biodegradable synthetic estrogen classes (Ting and Praveena, 2017). However, other birth control pill users naively flush these pills down the toilet drains (Cui et al., 2006; Ting and Praveena, 2017). Increased detection of synthetic estrogens in the aquatic systems poses health hazard risks as they can potentially interfere with the sexual reproduction of humans and other aquatic species. Health implications associated with estrogenic compounds in water include male fish feminization, altering reproductive characteristics and lowering sperm counts (Sumpter and Jobling, 2013; Du et al., 2020). Thus, oral birth control pills’ main ingredient (EE2) can alter entirely aquatic ecosystems since it can diminish the fish biomass (Sumpter and Jobling, 2013; Pinto et al., 2014). Human exposure to high estrogenic compound concentration is linked to prostate and breast cancer in men and women, respectively (Yang et al., 2011; Hallgren et al., 2014). Although the effects of estrogenic compounds on humans, plants, aquatic species and wildlife have been explored (Zhang et al., 2016; Adeel et al., 2017; Vilela et al., 2018), there is little information available in the literature regarding the effect of EE2 on bacterial communities autochthonous to the activated sludge.

The AS process is one of the most used biological (secondary) wastewater treatment processes in WWTPs. As a suspended-growth biological treatment process, AS rely on a dense microbial culture in suspension to biodegrade organic material under aerobic conditions and form a biological floc for solid separation in the settling units (Pell and Wörman, 2009). This process plays a pivotal role in wastewater treatment in producing contamination-free effluent before discharge into receiving waterbodies. The microbial community involved in AS assist in removing organic and inorganic contaminants (Samer, 2015). Previous studies have noted a decline in microbial abundance and richness in soil treated with co-mixture, including the natural form of EE2 (Zhang et al., 2014). A dehydrogenase activity test also revealed diminishing enzymatic activities among the soil microbial population. Thus, EE2 in the wastewater plant may present adverse effects, especially in the AS process where nutrient removal, biogas production, xenobiotic biodegradation, and recovery of other valuable resources occur. Despite the increased concentration of EE2 received by WWTPs, their effects on AS bacterial communities are not documented in the literature. Thus, the current study was tailored to investigate whether there is a shift in AS microbiome due to exposure to environmental and predictive elevated concentrations of EE2.

## Materials and methods

### Description of the study site, sample collection, and microbiome extraction

Glassware used in this study were washed and rinsed with methanol (MeOH), then dried before sample collection. During the study period, wastewater samples were collected from the Rooiwal Wastewater Treatment Plant (WWTP) in Pretoria, South Africa and transported to the Tshwane University of Technology (TUT) Water for analysis within 2 h of collection. The plant comprises three divisions: the first was established in the 1950s and the most recent in 1983, designed to treat a net flow of 245.3 Ml/d of water. The three divisions are Rooiwal West: 40.8 Ml/d (biological trickling filter facility), Rooiwal East: (biological trickling filter facility), and Rooiwal North (biological nutrient removal (BNR) AS plant). Approximately 300 g of AS was collected from the activated sludge compartment of the wastewater treatment plant in Pretoria and placed in a sterile container. Upon arrival to the laboratory, about 100 g of AS was gradually added into a sterile container containing 900 ml of 1× phosphate buffer solution (1×PBS) until the 1,000 ml mark was reached. The supernatant was filtered through a 3.0 μm filter (Sigma-Aldrich, South Africa) under the pressure of 2 bar to remove eukaryotic cells and debris and the resulting filtrates was then filtered through a sterile 0.22 μm membrane filter to capture bacterial cells. The filter papers were placed in a 2 ml microcentrifuge tube containing 1.5 ml of 1 × PBS and 20% of glycerol (Sigma-Aldrich, South Africa), then disrupted using a Disruptor Genie® Vortex mixer (Scientific Industries, South Africa) for 10 min. Each microfuge tube was then be centrifuged at 13,000× *g* for 1 min and the resulting microbial pellet were used as the experimental inoculum.

### Chemical acquisition

17 α-ethynylestradiol (EE2 or C_20_H_24_O_2_) 99% certified reference material standard used in this study was purchased from Minema (Johannesburg, South Africa). The rest of the chemicals were purchased from Sigma-Aldrich (Johannesburg, South Africa). The EE2 standard powder was dissolved in methanol and water at 50/50 (v/v). Prior to microbial inoculation, methanol was evaporated as described by Thiele-Bruhn and Beck (2005).

### Experimental setup

To assess the impact of EE2 on bacterial communities, 100 ml of modified minimal salt media was inoculated with a 1 ml of AS. The media was supplemented with carbon sources and nutrient supplements as follows: D-glucose anhydrous (2.5 g/l), MgSO_4_.H_2_O (0.5 g/l) and KNO_3_ (0.18 g/l). The pH of the applied media was adjusted to 7.2, using 1.0 M HCl and 1.0 M NaOH (Merck, South Africa). Experiments were performed using 250 ml Erlenmeyer flask as batch reactors at 35°C in a shaking incubator at 120 rpm for 6 days under aerobic conditions. The experimental study was divided into three series: (i) EE2-free (0 ng/L) media supplemented with AS, (ii) EE2 media supplemented with environmental (5, 10, and 100 ng/L) and (iii) predictive elevated (5, 10, and 100 mg/L) concentrations. The environmental concentrations used in this study were based on the ranges in which these estrogenic compounds are frequently detected in the environment between 1 and 100 ng/L (Pessoa et al., 2014; Kanama et al., 2018; Fang et al., 2019). The predictive elevated concentrations in the current study were based on the spillage of EE2 transit, which may dramatically increase the concentration to mg/L in the environment. Physicochemical parameters such as pH, dissolved oxygen (DO) and electrical conductivity (EC) were also assed during the study. As described below, a metagenomic approach combined with the latest generation sequencing platform and Mothur pipeline tools was used to identify and classify the sludge’s bacterial microbiome. Dehydrogenase activity analysis estimated total enzymatic activities in both non-incubated and incubated AS. All experiments were performed in triplicates.

### Response of culturable bacterial counts to EE2 stress

The viable microbial count was performed using a serial dilution method before and after incubation, according to APHA (2012). Different concentrations of EE2 working solutions were prepared at a total volume of 100 ml and mixed with 45°C nutrient agar media 900 ml before spreading onto Petri dishes. Briefly, 10 ml of wastewater samples was homogenized in 100 ml of buffered saline, and 10-fold dilutions were performed. Aliquots of 1 ml from the prepared serial dilutions were aseptically inoculated into modified nutrient agar (Merck) plates. The inoculated plates were then incubated at 37°C for 48 h. The bacterial counts were expressed as colony forming unit per milliliter (CFU/ml) described by APHA (2012). Each sample’s initial concentration was carefully recorded, while un-inoculated nutrient agar plates served as a control.

### DNA extraction, amplification, and sequencing of bacterial 16S rRNA genes

Microbial communities’ genetic material was recovered directly from inoculated into the reactors above, both non-incubated and incubated AS samples. Bacterial DNA was extracted using the ZymoBIOMICS DNA extraction Kit (Zymo Research, Pretoria, South Africa) according to the manufacturer’s instructions. The integrity and purity of extracted DNA were assessed using a Nanodrop spectrophotometer (NanoDrop 2000, Thermo Scientific, Japan). DNA sequencing was performed at Beijing Genomics Institute, a sequencing center. Upon arrival at the sequencing center (Beijing Genomics Institute), the extracted genomic DNA was assessed on the 1.0% agarose gel and Qubit 3.0 (Thermo Scientific, Japan). The 16S rRNA gene primers targeting specifically the V4 region (515F—5′-GTGCCAGCMGCCGCGGTAA 3′ and 806R—5′-GGACTACHVHHHTWTCTAAT 3′) were used to amplify the region of interest. Each PCR reaction mixture consisted of 50 of 25 μl of 2x Dream Taq™ PCR master mix (10 × Dream Taq™ buffer, 2 μM dNTP mix and 1.25 U Dream Taq™ polymerase), 21 μl nuclease-free water, 2 μl DNA template (50 ng/μL) and 1 μl (0.2 μM) of each primer pairs. The following PCR cycling parameters were used: initial denaturation step at 95°C for 5 min, followed by 30 cycles comprising of denaturation at 95°C for 40 s, annealing at 55°C for 2 min and extension at 72°C for 1 min, with a final extension step for 10 min at 72°C. The amplification PCR reaction was reported by Ekwanzala et al. (2019) was used, but with a slight modification on annealing temperature at 50°C. The triplicate samples were pooled in equimolar concentrations based on library concentrations and calculated amplicon sizes. The pooled PCR products were then shipped to and sequenced at Beijing Genomics Institute (BGI), Tai Po, Hong Kong, on the BGIseq-500 sequencing platform described by Ekwanzala et al. (2020) for paired-end reads of 300 bp long for each sample. Generated raw sequencing data were registered and deposited at the European Nucleotide Archive under accession number PRJEB38611 (https://www.ebi.ac.uk/ena/browser/view/PRJEB38611; ERP122046). Each concentration was submitted as a Biosample with accession numbers spanning SAMEA6869872 to SAMEA6869878.

### Bioinformatic analysis

All bioinformatic analysis was carried out on the Galaxy platform (Afgan et al., 2018). A community-driven data analysis protocol by previous investigators was followed to analyze the acquired 16S rRNA reads (Batut et al., 2018; Hiltemann et al., 2019). All functions required to implement the overall analysis pipeline are available within the Mothur software package (v.1.39.5) and are illustrated on the Mothur website (Schloss et al., 2009).^1^ Generated reads were first imported to the Galaxy platform.^2^ A list of dataset pairs was built to combine paired reads. These pairs were used to create contigs using the Make.contigs of the Mothur pipeline. Generated contigs between read pairs were filtered by length, quality and duplications using the Filter.seqs and Screen.seqs functions. Sequences were aligned against a reference alignment, and those sequences that did not align to the correct region were culled (Schloss, 2009, 2010; Schloss and Westcott, 2011). The sequences’ ends were trimmed so that the sequences started and ended at the exact alignment coordinates (Schloss, 2013). Unique sequences and their respective frequencies in each sample were identified and counted using the Unique.seqs and Count.seqs functions, respectively. A pre-clustering algorithm incorporated in the Pre.cluster function was used to denoise sequences within each sample (Schloss and Westcott, 2011). The resulting sequences were screened for chimeras using UCHIME (Edgar et al., 2011). A naive Bayesian classifier incorporated in Classify.seqs function was used to classify each sequence against the Ribosomal Database Project (RDP) 16S rRNA gene training set (version 16) customized to include rRNA gene sequences from mitochondria and Eukaryota. We required 0.20 cut-off as per Wang et al. (2007). The sequences that could not be classified to the kingdom level or those classified as Archaea, Eukaryota, chloroplasts or mitochondria were culled. Finally, sequences were split into groups corresponding to their taxonomy at the genus level and then assigned to operational taxonomic units (OTUs) at a 3% dissimilarity level, and rarefaction curves were also determined using Classify.otu and Rarefaction.single functions, respectively.

### Dehydrogenase activity analysis

The microbial activity of the sludge microbiome was evaluated using the dehydrogenase activity (DHA) test. The transformation of 2, 3, 5-triphenyl tetrazolium chloride (TTC, SIGMA, SA) to 1, 2, 5-triphenyl formazan (TPF) was employed as previously reported (Zhang et al., 2014). Prior to application, the collected fresh sludge was filtered through a 3.0 μm filter (Sigma-Aldrich, South Africa) to remove excess moisture, eukaryotic cells and debris. Briefly, 5 ml of 0.3% of TTC solution and 0.1 M of tris (hydroxymethyl) aminomethane buffer adjusted with 32% of HCl to pH 7.2 were added to 5 g sludge in a 50 ml centrifuge tube. After an incubation of 24 h at 37°C in the dark, a few drops of H_2_SO_4_ were added to interrupt the reaction. To blank samples, no TTC solution was added. The product, TPF, was extracted with 5 ml of toluene on a horizontal rotary shaker. Subsequently, the solution was centrifuged for colorimetric determination at 546 nm. The dehydrogenase activity was reported as micrograms TPF per gram of dry weight/day (TPF gdw/day).

### Data and statistical analysis

All the experiments conducted in this study were carried out in triplicates. Thus, the figures in this study represent mean values ± standard deviations. The Shannon diversity index (H′) and the Chao 1 species richness estimator were determined to estimate each water sample’s microbial diversity and richness for metagenomics analysis. The relative abundance of individual taxa within each community was calculated by comparing the number of sequences assigned to a specific taxon against the number of sequences obtained for that sample. The similarity and dissimilarity in bacterial community structure within the control and EE2-supplemented laboratory batch reactors were analyzed using the Jaccard and the Yue and Clayton theta index. This information was used to assess microbial fluctuation by comparing untreated and treated samples. As described elsewhere, the rarefaction curves were also determined (Cole et al., 2009). The relative abundance (%) of the top 10 OTUs at phylum, class, order, family, genus, and species taxa level for each concentration was displayed using Phinch2 visualization framework v.2.0.1 (Bik, 2014). Finally, the analysis of variance (ANOVA) test using GraphPad Prism version 8.0.0 for Windows (GraphPad Software, San Diego, California, United States) was used to analyze the variance induced by EE2 on the cultured-dependent and culture-independent of the microbiome of AS.

## Results

### Effect of EE2 on physicochemical characteristics of wastewater

The AS samples from this study were subjected to physicochemical analyses focusing on parameters known to influence bacterial diversity and activity, such as pH, DO, and EC. Table 1 summarizes the fluctuations in physicochemical parameters observed in the untreated and treated samples following a 6-day incubation at different EE2 concentrations. Overall, the physicochemical parameters tested during the experimental study demonstrated an increase in the values from untreated to treated samples. All the samples (both treated and untreated) had a neutral pH, ranging between 6.9 and 7.38, following a 6-day incubation period. Regarding the DO, the control showed a higher DO compare to EE2 spiked samples after incubation. The temperature was kept at 35°C throughout the experimental study.

**Table 1:** Physicochemical characteristics of control and treated samples following a 6-days incubation at different EE2 concentrations.

EE2 concentrations	pH		EC (S/m)		DO (mg/L)	
	Initial	Final	Initial	Final	Initial	Final
0 ng/L	6.9	7.25	6.35	8.55	8.16	7.8
5 ng/L	6.9	7.10	6.35	7.90	8.16	6.0
10 ng/L	6.9	7.15	6.35	7.91	8.16	7.2
100 ng/L	6.9	7.09	6.35	7.78	8.16	7.0
5 mg/L	6.9	7.00	6.35	7.50	8.16	7.6
10 mg/L	6.9	7.32	6.35	7.45	8.16	6.91
100 mg/L	6.9	7.38	6.35	7.44	8.16	6.89

### Effect of EE2 on viable bacterial counts of wastewater

**Figure 1:**
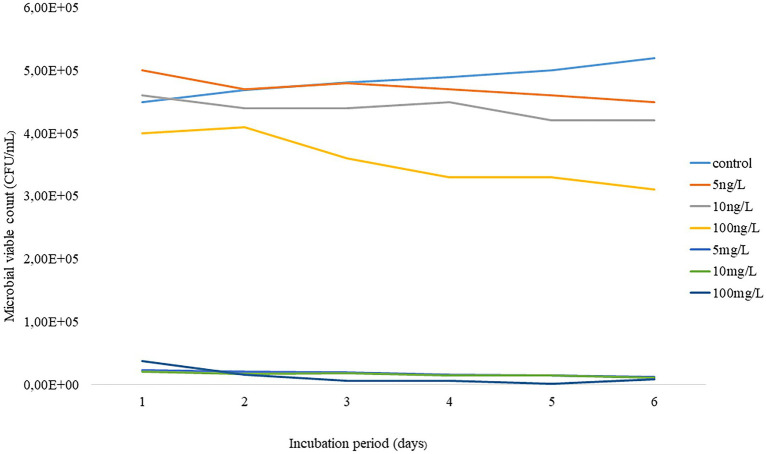
Temporal trends of AS viable bacteria under predictive elevated concentrations (5 – 100 mg/L) and environmental concentrations (5 – 100 ng/L) of EE2 over a 6-day incubation.

The shift in viable bacterial counts was evaluated by enumerating the bacteria concentrations before and after the 6-day incubation, as indicated in Figure 1. The initial count of the AS microbiome was 56 × 10^5^ CFU/ml, which slightly decreased to 45 × 10^5^ CFU/ml at the end of the experiment. Interestingly, no significant shift was observed in the richness of AS viable bacterial counts under environmental concentration treatment. In contrast, predictive elevated concentrations during the incubation period resulted in a notable bacterial change from day 1. The current study demonstrated that about 10^2^ CFU/ml was lost across all test concentrations following a 24 h incubation. Only about 8 × 10^2^ CFU/ml remained after 6 days of incubation at 100 mg/L.

### Microbial community structure in the presence of EE2

**Figure 2:**
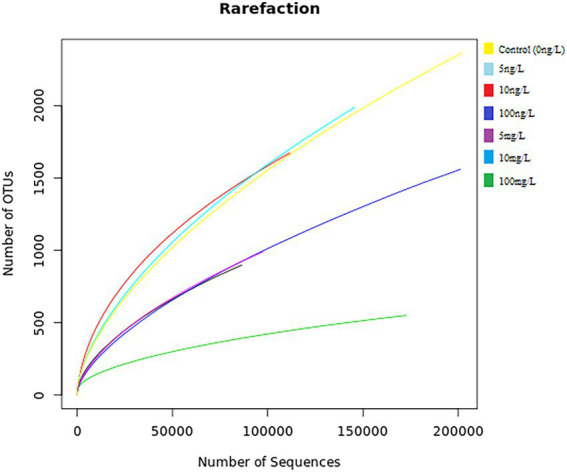
Rarefaction curves for the seven samples [control 0 ng/L EE2 (yellow), 5 ng/L EE2 (light blue), 10 ng/L EE2 (red), 100 ng/L EE2 (dark blue), 5 mg/L (purple), 10 mg/L (blue), and 100 mg/L (green)] showing the coverage depth as compared to the total number of sequences. The x-axis represents the number of sequences sampled while the y-axis represents the number of OTUs. The legend on the top right indicates each color coded to its respective concentration.

The seven 16S metagenomic samples sequenced using a BGISEQ-500 platform generated more than 276 Mb of zipped data accounting for 1,361,64 sequences (an average of ~194,521 sequences per sample). In total, 1,017,038 high-quality DNA sequences were processed, and an average of 145,291 was found from each sample. The plotted rarefaction curve revealed that analysis supported the 86,463 sequences, which were enough to cover the full depth of the microbial diversity and richness of the sampled concentrations (Figure 2).

**Figure 3:**
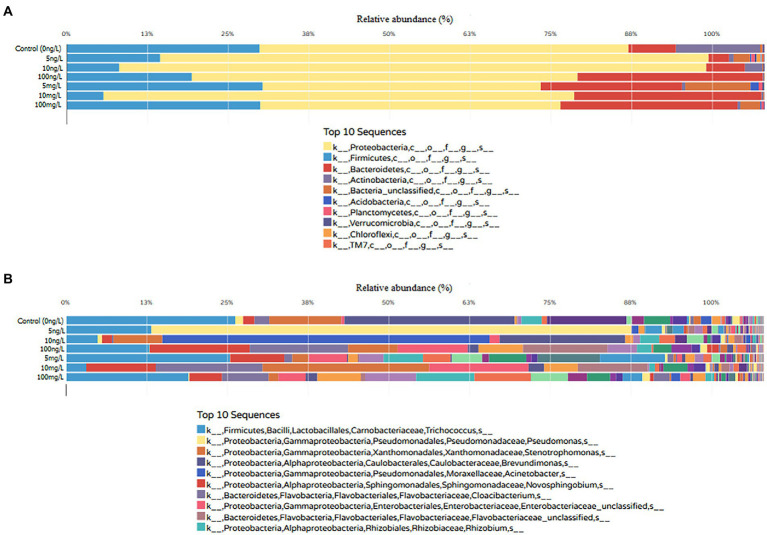
Relative abundance and phylum-level **(A)** and genus-level **(B)** taxonomic classification of 16S rRNA amplicons across sequenced samples [environmental (5, 10, and 100 ng/L) and predictive elevated concentrations (5, 10, and 100 mg/L)] against the SILVA prokaryotic reference database. The x-axis shows the different EE2 concentrations while the y-axis shows the relative abundance in percentage of identified phylum and genus of the top 10 sequences.

The taxonomic classification among concentrations revealed significant differences in the community composition between control (0 ng/L), environmental concentrations (5, 10, 100 ng/L) and predictive elevated concentrations (5, 10, and 100 mg/L; *t*-test, all *p* < 0.05). At the phylum level, 23 phyla were identified in bioreactors containing EE2. Among the 23 bacterial phyla identified, the most frequently detected, as displayed in Figure 3A, were *Proteobacteria* (ranging from 40% to 84% across all EE2 supplemented bioreactors), *Firmicutes* (ranging from 5% to 28% across all EE2 supplemented bioreactors) and *Bacteroidetes* (ranging from 3% to 27% across all EE2 supplemented bioreactors). Unclassified reads accounted for 0.06% to 9%. Low abundance phyla included *Acidobacteria*, *Actinobacteria*, *Armatimonadetes*, Chlamydiae, *Chlorobi*, *Chloroflexi*, BRC1, *Deferribacteres*, *Deinococcus*-*Thermus*, *Elusimicrobia*, *Fibrobacteres*, *Fusobacteria*, *Gemmatimonadetes*, *Lentisphaerae*, *Nitrospira*, *Planctomycetes*, *Spirochaetes*, *Synergistetes*, *Thermotogae*, *Verrucomicrobia*, and TM7.

At the genus level Figure 3B, the BGISEQ-500 sequencing platform detected 408 bacterial genera in the EE2 supplemented bioreactor. Among the 408 genera identified, members from the *Trichococcus*, *Pseudomonas*, *Stenotrophomonas*, *Brevundimonas*, *Acinetobacter*, *Novosphingobium*, *Cloacibacterium* and *Rhizobium* were found to be the dominant genera in EE2 supplemented bioreactor. Results at class, order and family levels are shown in Supplementary Figures S1–S3.

**Figure 4:**
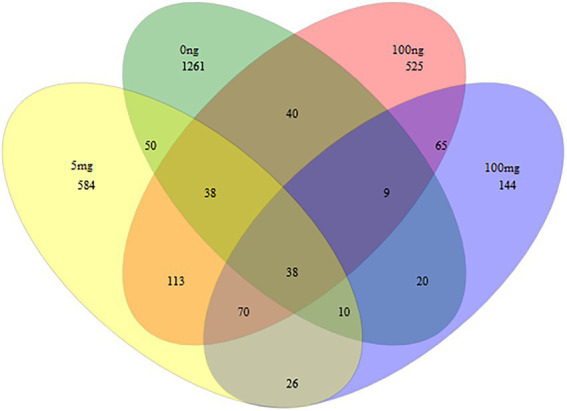
A Venn diagram depicts the overlap (or shared) of most abundant species in a multidimensional network between 0 ng/L, 100 ng/L, 5 mg/L, and 100 mg/L of EE2. The number inside overlapping ellipses denotes shared species between concentration treatments. We observed 1,056, 152, 239, and 0 unique species for 0 ng/L, 100 ng/L, 5 mg/L, and 100 mg/L of EE2, respectively.

A further analysis was conducted using a Venn diagram (Figure 4) to reveal the shared microbial OTU that survived the effect of EE2 on activated sludge. We picked four concentrations, i.e., 0 ng/L, 100 ng/L, 5 mg/L, and 100 mg/L. Thirty-eight OTU were shared between these assessed concentrations. The full list of all shared OTU is shown in Table 2. However, when analyzing the proportion of increased or decreased percentages as depicted in Table 2, only seven OTUs *Novosphingobium* (OTU0003)*, Cloacibacterium* (OTU0004)*, Stenotrophomonas* (OTU0005), Enterobacteriaceae_unclassified (OTU0007), *Stenotrophomonas* (OTU0034), *Enterobacteriaceae*_unclassified (OTU0049) and Rhodobacteraceae_unclassified (OTU0063). *Stenotrophomonas* (OTU0005 and OTU0034) had the most remarkable increase, with an average of more than 738.43% increase. This occurrence was followed by two unclassified *Enterobacteriaceae* (OTU0007 and OTU0049) and *Rhodobacteraceae* (OTU0063) with an average of 253.49 and 128.57% increase, respectively. A slight increase was observed for *Cloacibacterium* and *Novosphingobium* with a 12.07% and 5.08% increase, respectively. All other OTUs were reduced by 18.75% to 98.60% except for OTU, which proportion remained the same before and after treatment.

**Table 2:** Proportion of the 38 shared OTUs between 0 ng/L and 100 mg/L concentrations and highlighting an increased or decreased percentage.

OUT number	Genera	Non-incubated abundance	Abundance at 0 ng/L	Abundance at 100 mg/L	Increase or decrease percentage
Otu0001	*Trichococcus*	34,733	34,726	2,737	−92.11
Otu0003	*Novosphingobium*	9,290	9,264	9,735	5.08
Otu0004	*Cloacibacterium*	13,119	13,109	14,692	12.07
Otu0005	*Stenotrophomonas*	2,668	2,648	22,111	735.00
Otu0007	*Enterobacteriaceae*_unclassified	7,499	7,455	13,288	78.24
Otu0008	*Brevundimonas*	1,036	1,024	172	−83.20
Otu0009	*Comamonadaceae*_unclassified	12,730	12,709	4,651	−63.40
Otu0011	*Leuconostoc*	14,569	14,448	201	−98.60
Otu0013	*Rhizobium*	8,281	8,261	354	−95.71
Otu0014	*Rhizobium*	6,673	6,665	299	−95.51
Otu0023	*Rhodobacteraceae*_unclassified	2,675	2,664	1,011	−62.04
Otu0024	*Pseudoxanthomonas*	1858	1847	603	−67.35
Otu0028	*Lactobacillales*_unclassified	1,691	1,683	506	−69.93
Otu0032	*Azotobacter*	1,044	1,052	85	−91.92
Otu0034	*Stenotrophomonas*_unclassified	130	129	1,086	741.86
Otu0035	*Brucella*	560	491	211	−57.02
Otu0040	*Alcaligenaceae*_unclassified	690	677	419	−38.10
Otu0049	*Enterobacteriaceae*_unclassified	97	87	460	428.73
Otu0062	*Rhodobacter*	553	521	97	−81.38
Otu0063	*Rhodobacteraceae*_unclassified	166	147	336	128.57
Otu0070	*Arthrobacter*	699	499	30	−93.98
Otu0076	*Massilia*	9	6	1	−83.33
Otu0079	*Bacteroides*	950	843	168	−80.07
Otu0114	*Methylobacterium*	16	4	1	−75
Otu0121	*Leuconostoc*	55	42	3	−92.85
Otu0127	*Pleomorphomonas*	163	141	28	−80.14
Otu0128	*Microbacteriaceae*_unclassified	28	22	5	−77.27
Otu0140	*Microbacteriaceae*_unclassified	98	79	6	−92.40
Otu0144	*Trichococcus*	89	79	9	−88.60
Otu0149	*Xanthobacter*	84	64	18	−71.87
Otu0163	*Roseomonas*	43	35	19	−45.71
Otu0175	*Paracoccus*	11	8	1	−87.5
Otu0180	*Rhizobiales*_unclassified	35	29	1	−96.55
Otu0198	*Rhizobiales*_unclassified	88	70	13	−81.42
Otu0226	*Corynebacterium*	32	16	13	−18.75
Otu0240	*Lactobacillales*_unclassified	21	18	10	−44.44
Otu0242	*Paracoccus*	44	32	4	−87.5
Otu0265	*Microbacteriaceae*_unclassified	22	11	11	0

### Community species richness and diversity indices

The microbial alpha diversity was evaluated based on 86,463 sequences randomly selected from each sample. According to the calculated Shannon index, bioreactors with control and environmental concentrations (5, 10, and 100 ng/L) had the most diverse microbial community, with Shannon indexes ranging from 2.58 to 3.68. In contrast, the predictive elevated EE2 concentrations showed lower microbial diversity with Shannon indexes ranging from 2.24 to 2.84. Environmental concentrations were not statistically different among themselves (*t*-test, *p* > 0.05) and likewise for the predictive elevated concentrations (*t*-test, *p* > 0.05). However, all environmental concentrations were statistically (*t*-test, *p* < 0.05) different except for 10 ng/L and 10 mg/L (*t*-test, *p* > 0.05). Chao indexes also showed the same pattern as the Shannon indexes (see Supplementary Figure S4).

The microbial beta diversity, which measures the similarity of the membership and structure between different samples, was also evaluated based on 86,463 sequences randomly selected from each sample through its Yue and Clayton theta similarity coefficient and Jaccard index (Supplementary Figures S5A,B). According to the Jaccard index, environmental concentrations among themselves showed a darker red, indicating the high similarity in the membership and structure found in those samples. However, light red indicated a dissimilar microbial community and structure between the samples. Likewise, the Yue and

Clayton theta similarity coefficient showed a similar pattern with darker red, emphasizing the differences between community membership and the structure of environmental and predictive concentrations. We further generated dendrograms to better relate the samples’ similarities to each other. The dissimilarity among environmental and predictive concentrations with few exceptions, especially for the Yue & Clayton theta similarity coefficient dendrogram, is shown in Supplementary Figures S4C,D.

### Effect of EE2 on enzymatic activity within AS

**Figure 5:**
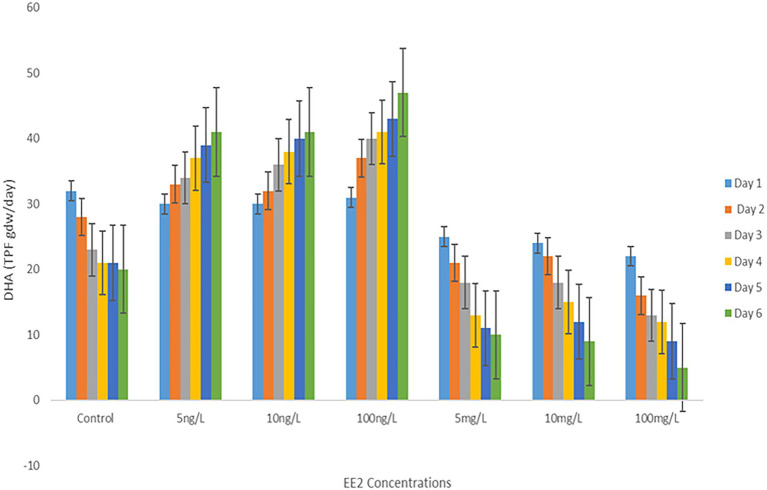
Comparison of DHA of AS treated with different concentrations of EE2 during a 6-day incubation.

The effect of EE2 on the AS total enzymatic activity was evaluated by monitoring the DHA of the microbial population during incubation in different EE2 concentrations. Notably, there was a downward trend in DHA activity, as depicted in Figure 5. During the experiment, less enzyme activity loss was observed in samples incubated with environmental EE2 concentrations ranging between 5 and 100 ng/L during the experiment. Nevertheless, the predictive elevated concentrations of EE2 reduced enzyme activity significantly; about 40% of DHA was lost at 100 mg/L by the end of day 6.

## Discussion

The physicochemical parameters are of paramount importance for the well-being of the water environment and aquatic life. The hydrogen-ion concentration is an imperative quality parameter for wastewaters to describe wastewater’s acidic or basic properties. A pH less than 7 in wastewater indicates septic conditions, while values less than 5 and greater than 10 indicate the presence of industrial wastes and non-compatibility with biological operations (Edokpayi et al., 2017; Aniyikaiye et al., 2019). The pH concentration range for the existence of biological life is relatively narrow, typically 6–9 (Hellawell, 2012). As demonstrated in Table 1, there was a slight increase in pH in predictive elevated EE2 concentrations. However, the pH still ranged between 8.10 and 8.38, which is within the WHO guidelines for wastewater discharge (WHO, 2011). Furthermore, it was shown by Bhateria and Jain (2016) pH levels beyond the 6.5 to 9.5 can damage and corrode pipes and other systems, raising heavy metal toxicity even further. A slight increase in pH can lead an oligotrophic (low dissolved oxygen) lake to become eutrophic (lacking dissolved oxygen). Minor pH changes can have long-term consequences (Bhateria and Jain, 2016). Future studies should be directed at understanding the long-term impacts of EE2 on the pH of wastewater to understand the extent to which it increases hydrogen ion concentrations under natural conditions within WWTPs. Extreme pH in wastewater damages biological processes in biological treatment units (Grady et al., 2011; Cai et al., 2014).

Another parameter that has a significant effect on the characteristics of microbial medium is DO. It is an important parameter in that, during the actual wastewater process, air is forced into the aeration basins and increases the activity of these microorganisms in addition to aids in the mixing of organic waste. In general, an increase in the values of physicochemical variables was observed except for DO, which showed an approximately 40% decrease across all samples. This could be due to the growth stimulation of some microorganisms, which inevitably led to oxygen consumption during the study period. The decrease in samples spiked with predictive elevated EE2 concentrations could be attributed to the negative impact of EE2 on bacterial growth and biomass production. The lower concentrations of DO negatively affect the successful conversion of organic wastes into non-toxic state due to scarcity of oxygen for aerobic microorganisms (Basile et al., 2011; Meli et al., 2016).

The AS microcosms were prepared using synthetic estrogen EE2 concentrations at 0 ng/L (used as control) 5, 10, and 100 ng/L for environmental and 5, 10, and 100 mg/L for high predictive concentrations to investigate the effect on AS microbiome. Unlike other environmental synthetic estrogens, EE2 is not easily biodegraded within the WWTPs, and therefore it ends in the environment (Zhang et al., 2016). Furthermore, it is reported that most synthetic estrogen de-conjugate after the early stages of wastewater treatment increases the estrogenic compound concentration in the wastewater (Marti and Batista, 2014). This information suggests that EE2 is present in the secondary stage of AS within the WWTPs. Its success depends on microbial richness and diversity in the reactor; its presence in high concentration causes a negative shift in the AS microbiome, as revealed in this study. Thus, processes such as biological nutrient removal (BNR), which occur in the reactor and are essential for removing phosphates and ammonia in wastewater, may be impacted negatively due to the presence of EE2 in high concentrations. Previous investigators (Kamika and Momba, 2013; Mboyi et al., 2017) have shown that a consortium of bacterial species, autochthonous to wastewater, could remove inorganic pollutants, such as phosphate and ammonia and attributed their proficiency to the richness of the biomass. Thus, it is crucial to remove these estrogenic compounds before biological processes are applied in a real situation in wastewater treatment as EE2 shows to be detrimental to wastewater biomass, which inevitably hinders all the processes dependent on biomass richness. Notably, there were 8 × 10^2^ CFU/ml, which remained following incubation with EE2 (100 mg/L) for the duration of the experiment, signaling those viable cells from AS may be useful in the clean-up of EE2 contaminated environments during spillage due to their ability to mineralize EE2. However, since EE2 has been found in ng/L concentrations in the environment, the current study reveals that an estimated bacterial range of 31–45 × 10^4^ CFU/ml possesses the ability to survive and possibly degrade environmental concentrations of EE2. Although EE2 generally has detrimental effects on the general microbial population of AS, this source may be a potential reservoir for estrogenic compound degrading bacterial agents and needs further exploration. Furthermore, it has been reported that human gastrointestinal tract bacteria contribute to 12%–15% of the 16S rRNA gene of the bacterial microbiome within the WWTPs (Cai et al., 2014; Su et al., 2015). As revealed in the current study, EE2 concentrations as high as 5 mg/L caused a significant shift in the AS microbiome, suggesting that consuming high dosage of EE2 in oral birth control pills may cause changes in the human gastrointestinal tract microbiome. Previous studies have demonstrated that the consumption of NSAIDs (non-steroidal anti-inflammatory drugs) in high dosage might result in mild to life-threatening side effects (Newton et al., 2015). This enteropathy was attributed to the shift in small intestine bacterial richness and diversity (Fisher et al., 2015; Shchegolkova et al., 2016).

Metagenomics analysis revealed notable bacteria community diversity was mostly apparent in lower taxonomic levels. The bacterial community diversity slowly decreased as the EE2 concentrations increased, as depicted in Figure 3A. Non-EE2 spiked samples maintained a higher diversity than those spiked with various concentrations of EE2 in terms of phylum, classes, orders, families and genera. Twenty-three phyla were identified in EE2 spiked samples, with *Proteobacteria* being the most predominant (accounting for 40% to 84%) across different concentrations. Other dominant phyla noted in EE2 spiked samples were Firmicutes (accounting for 5% to 28%) and Bacteroidetes (accounting for 3% to 27%). This study noted that EE2 at both environmental and predictive elevated concentrations could drastically change the bacterial community from phyla down to species level. These findings affirmed that AS microbial community is sensitive to heightened concentrations of EE2 in the environment. Shannon index showed that samples spiked with environmental EE2 concentrations had the most diverse microbial community, with Shannon indexes ranging from 2.58 to 3.68. In contrast, the predictive elevated concentrations showed lower microbial diversity with Shannon indexes ranging from 2.24 to 2.84.

Generally, the decrease in the number of reads was found to be inversely proportional to the increase in EE2 concentrations, as shown in Figure 3A. Despite a gradual reduction due to increased EE2 concentrations, the phylum *Proteobacteria* remained dominant across all samples suggesting resistance to selective pressures of EE2. The four predominant phyla identified in this study have previously been the most critical in estrogenic compounds’ bioremediation in wastewater (Budeli et al., 2020). These phyla aid in guiding researchers for bioprospecting potent estrogenic compounds degrading species. A systematic review conducted by Budeli et al. (2020) revealed that 60% of the estrogenic compound-resistant bacteria isolated in the past 15 years belonged to *Proteobacteria* phylum, suggesting that it is a current research hotspot and necessitates further investigations. These findings were consistent with Zhang et al. (2016), who demonstrated a positive correlation between estrogenic compound removal with *Proteobacteria* as the main functional bacterial phylum during cometabolic degradation of estrogenic compound. Furthermore, the microbial ecology of 14 wastewater treatment plants across China confirmed that *Proteobacteria* was the most abundant phylum in wastewater, constituting about 21% to 53% of the bacterial population in the wastewater treatment plants (Wang et al., 2012).

Moreover, about 0.06% to 9% were assigned as unclassified reads. These findings highlight many other sequences that could not be assigned to specific taxa, further affirming that we have only scratched the surface for novel bacterial species to be explored for estrogenic compound degradation and other biotechnological applications in wastewater. It is also worthy of note that Wang et al. (2012) did not record some bacterial genera detected in this study, such as *Deinococcus-Thermus*, *Thermotogae Planctomycetes* and *Chlorobi,* suggesting that wastewater bacterial microbiome varies from one another depending on the location and country. At genus level, members from the *Trichococcus, Pseudomonas, Stenotrophomonas, Brevundimonas, Acinetobacter, Novosphingobium, Cloacibacterium*, and *Rhizobium* were found to be the dominant genera in EE2 environmental samples.

Interestingly, some bacterial genera are used in EE2 bioremediation studies (Budeli et al., 2020), such as *Brevundimonas, Acinetobacter*, and *Novosphingobium.* Other bacterial isolates that are known to be proficient in EE2 bioremediation, such as *Rhodococcus, Sphingobium*, and *Acinetobacter,* were found in low concentrations. Although not directly related to the current investigation, the shift of wastewater bacterial community at different levels under environmental concentrations pressures highlights the need to investigate the gut microbiome response to EE2 since 97% of fecal taxa are represented in the sewage (Fisher et al., 2015; Newton et al., 2015; Ahmed et al., 2020). Thus, studies investigating the association between the high dosage of EE2 and the intestinal microbiome are vital to ascertain the side effects of high EE2 concentration consumption/exposure.

It is also important to note that the enzyme activity could not ultimately decrease even at the highest concentration of EE2, as an activity of DHA could still be detected after 6 days of the experiment at 100 mg/L, further proving that AS harbor potent EE2 mineralizing bacteria, as reported by (Zhang et al., 2016). The literature reported few bacterial isolates to degrade EE2 to a non-toxic state. Thus, future studies should also focus on identifying bacterial species demonstrating high enzymatic activities under high concentrations of EE2, as this would contribute to EE2 degrading the bacteria gene pool. Bacterial enzymes have been proven to have a powerful ability to degrade a wide range of micro-pollutants such as phenolic compounds, aromatic heterocyclic compounds and amine-containing aromatic compounds (Beck et al., 2018). In addition, wastewater bacterial enzymes are known to possess the ability to remove organic substrate electrons and ultimately reduce dioxygen molecules of micro-pollutants in the aquatic system (Unuofin et al., 2019). Hence, a decline in total enzymatic activity will negatively impact biological processes dependent on bacteria’s enzymatic ability in wastewater. Although there is no study directed at the effect of EE2 on microbial populations, Zhang et al. (2014) reported that a natural form of EE2 (E2) could enhance the enzyme activity of soil microbial populations. In the same, it was noted that microbial populations increased with the increase in E2 concentrations. The apparent downward trend in this study with the rise in EE2 concentrations may be attributed to EE2, among other estrogenic compounds being the most recalcitrant to biodegradation. Microorganisms can degrade the pollutant’s parental compound and utilize the metabolites for their metabolic activities, hence increasing microbial populations during bioremediation. However, when the pollutants are recalcitrant to bioremediation, such as EE2, microorganisms are left with no source of carbon and nitrogen necessary for their metabolic activities, hence the decline in microbial populations. The diminishing of enzymatic activity as a result of EE2 spiked samples is similar to the effect of other bioremediation recalcitrant compounds (Kamika and Tekere, 2017; Xu et al., 2019). Thus, although natural environmental estrogens are known to have stimulatory effects on microbial populations, the existence of EE2 as a co-pollutant is most likely to reverse those effects, as evidenced by the findings of the current study. In contrast, lower concentrations of EE2 (5–10 ng) promote richness of microbiome and dehydrogenase activity of AS during the course of the study as shown in Figures 2, 5, respectively. These findings suggest a potential occurrence of hormesis, which is defined as a phenomenon where xenobiotics confer stimulatory effects in a biological cell when administered in lower doses. The biological response to low levels of toxins and other stressors is generally favorable within the hormetic zone (Kim et al., 2018; Skaperda et al., 2021). However, further studies should investigate the combined effects of environmental estrogenic compounds and other EDCs. Therefore, removing residual pollutants, particularly EE2 in wastewater, is strongly suggested to ensure sludge microbial ecological safety.

## Conclusion

The present study enhances our understanding of the AS microbial community’s response to environmental and predictive elevated concentrations of EE2. Overall, the results of this study reveal that a higher concentration of EE2 causes a significant threat to the biological sludge microbial community. Therefore, removing residual pollutants, particularly EE2 in wastewater, is strongly suggested to ensure sludge microbial ecological safety. In addition, findings obtained in this study suggest a potential occurrence of hormesis as evidenced by promotion of microbiome richness and dehydrogenase activity of AS in lower EE2 doses (5–100 ng/L). However, further research addressing the long-term effects of EE2 in the sludge process is required to ascertain these claims. More studies are needed to include a broader range of environmental estrogenic compounds and a longer incubation time to mimic the actual AS process.

## Data availability statement

The datasets presented in this study can be found at the European Nucleotide Archive under accession number ERP122046 or PRJEB38611 (https://www.ebi.ac.uk/ena/browser/view/ PRJEB38611).

## Author contributions

PB: conceptualization. PB, ME and MM: data curation, formal analysis, and investigation. MM: funding acquisition, project administration, resources, supervision, and writing—review and editing. PB, ME, and MM: methodology. ME: Software. PB and ME: validation, visualization, and writing—initial draft. All authors contributed to the article and approved the submitted version.

## Funding

This research article received funding from the Department of Science of Technology and National Research Foundation, South Africa, through the SARChI Chair in Water Quality and Wastewater Management (grant number: UID87310) and the Tshwane University of Technology, South Africa. PB was funded by NRF Freestanding, Innovation, Scarce Skill Development Fund and South Africa (grant number: UID108484).

## Conflict of interest

The authors declare that the research was conducted in the absence of any commercial or financial relationships that could be construed as a potential conflict of interest.

## Publisher’s note

All claims expressed in this article are solely those of the authors and do not necessarily represent those of their affiliated organizations, or those of the publisher, the editors and the reviewers. Any product that may be evaluated in this article, or claim that may be made by its manufacturer, is not guaranteed or endorsed by the publisher.

## Author disclaimer

Opinions expressed and conclusions arrived at are those of the authors and are not those of the funders.

## Footnotes

^1^ http://www.mothur.org/wiki/MiSeq_SOP

^2^ usegalaxy.com
