# Supplementary material for: Hormetic effect of 17α-ethynylestradiol on activated sludge microbial community response
Source: Front Microbiol. 2022 Aug 18;13:961736. doi: 10.3389/fmicb.2022.961736 (PMC9434213; doi:10.3389/fmicb.2022.961736)
Supplement: Supplementary file 1 [file Presentation_1.pdf]

Supplementary information for Budeli et al.

EE2 impacts on AS' microbiome.

Phumudzo **Budeli**[1] • Mutshiene Deogratias **Ekwanzala**[2,3] • Maggy Ndombo Benteke **Momba**[1]*

[1] Department of Environmental, Water and Earth Sciences, Tshwane University of Technology, Arcadia Campus, Private BagX680, Pretoria 0001, South Africa.

[2] Centre for Antibiotic Resistance Research (CARe), University of Gothenburg, 41346 Gothenburg, Sweden

[3] Department of Infectious Diseases, Institute of Biomedicine, University of Gothenburg, 41346 Gothenburg, Sweden

✉ MNB Momba, Tel: +27123826365; E-mail: mombamnb@tut.ac.za

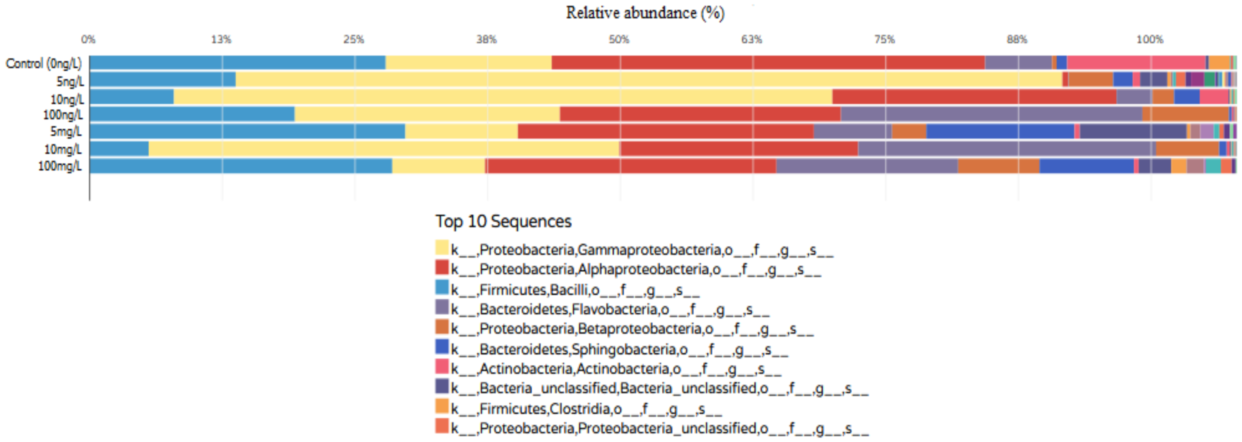

Fig. S1 Relative abundance and class-level taxonomic classification of 16S rRNA amplicons across sequenced samples [environmental (5, 10 and 100 ng/L) and predictive elevated concentrations (5, 10 and 100 mg/L)] against the SILVA prokaryotic reference database.

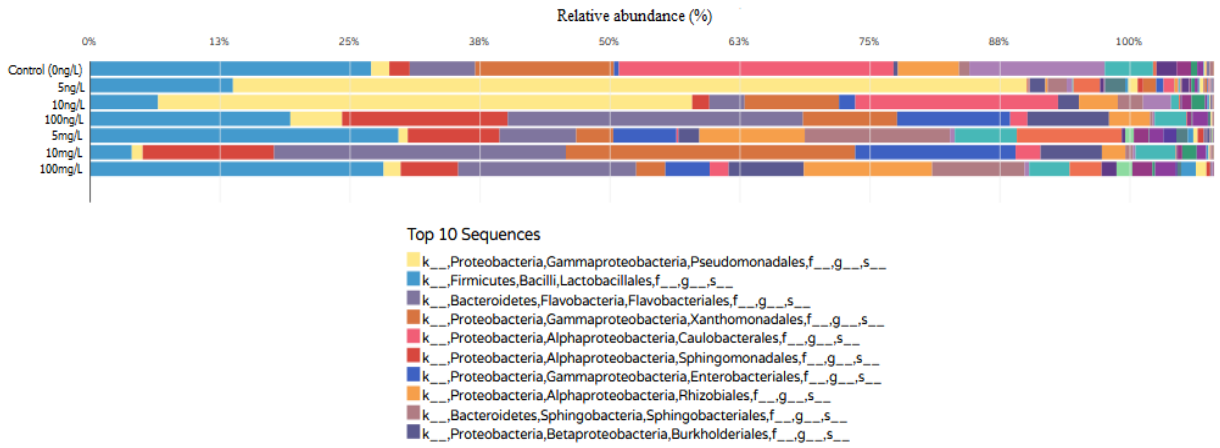

Fig. S2 Relative abundance and order-level taxonomic classification of 16S rRNA amplicons across sequenced samples [environmental (5, 10 and 100 ng/L) and predictive elevated concentrations (5, 10 and 100 mg/L)] against the SILVA prokaryotic reference database.

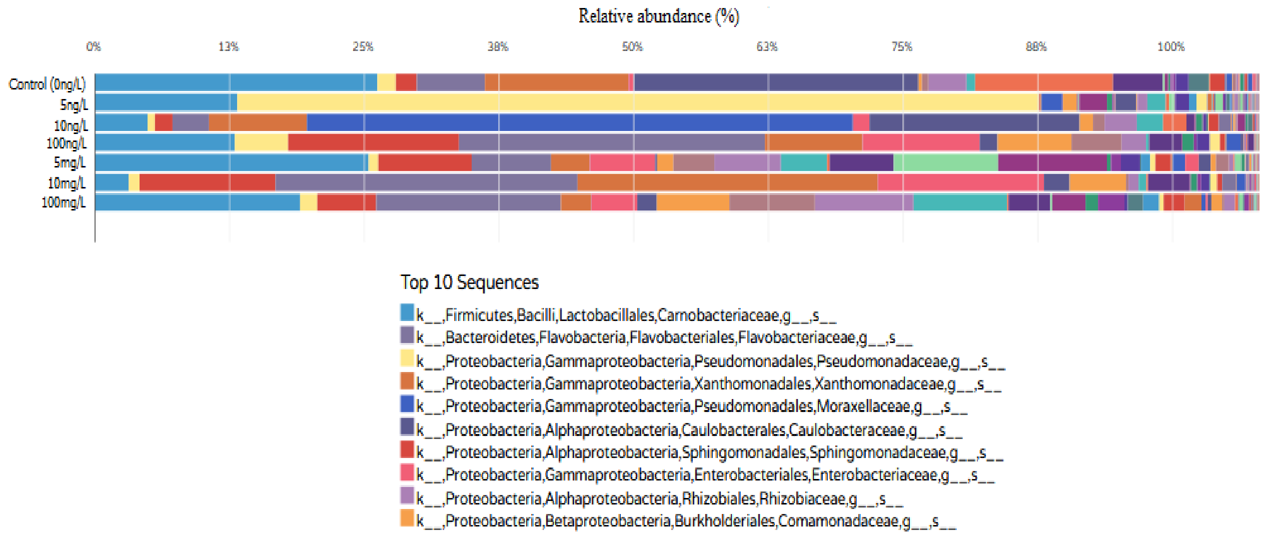

Fig. S3 Relative abundance and family-level taxonomic classification of 16S rRNA amplicons across sequenced samples [environmental (5, 10 and 100 ng/L) and predictive elevated concentrations (5, 10 and 100 mg/L)] against the SILVA prokaryotic reference database.

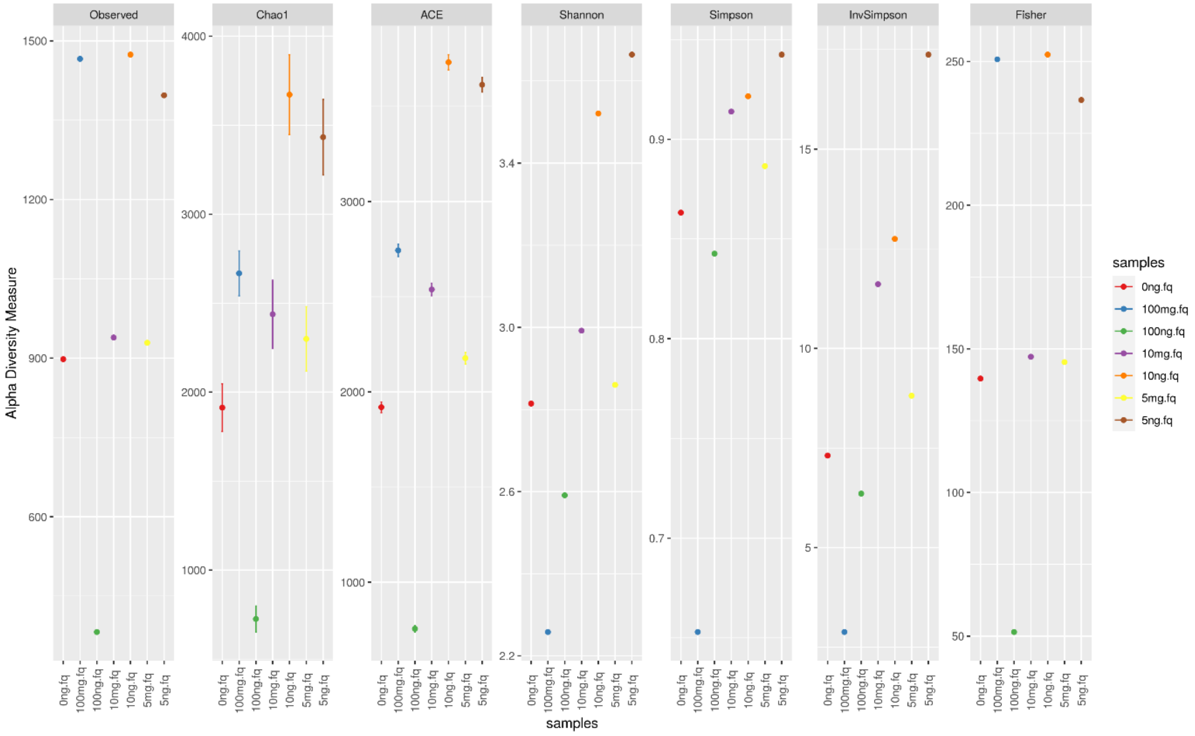

Figure S4 Diversity indices.

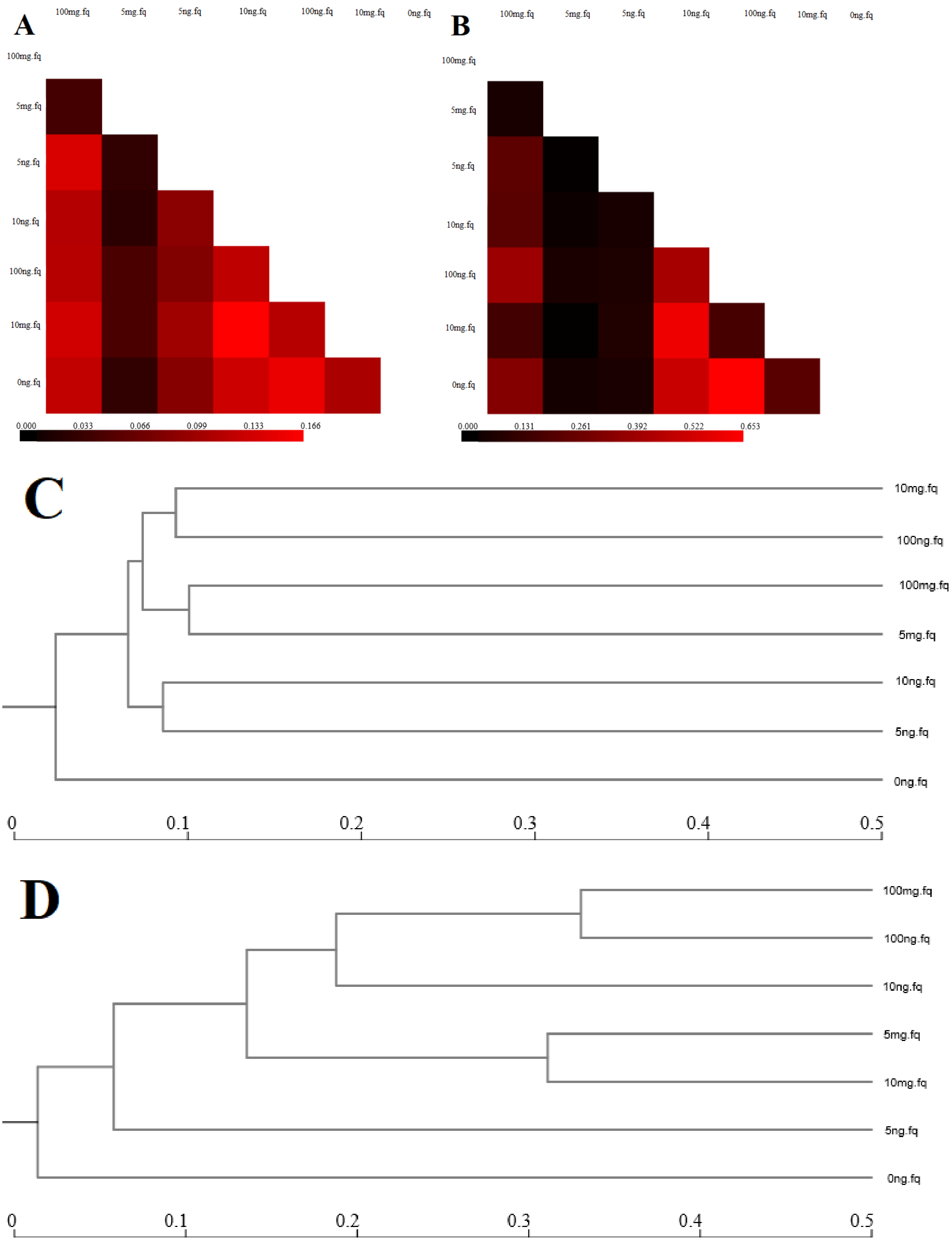

Figure S5 Yue & Clayton theta (A and B) and Jaccard indices (C and D) similarity metrics was used to assess the differences across microbial communities.
